# Antibacterial constituents of *Fructus Chebulae Immaturus* and their mechanisms of action

**DOI:** 10.1186/s12906-016-1162-5

**Published:** 2016-07-02

**Authors:** Kun Li, Yue Lin, Bin Li, Taowen Pan, Fei Wang, Ruqiang Yuan, Jianjun Ji, Yunpeng Diao, Shouyu Wang

**Affiliations:** College of Chemistry and Chemical Engineering, Liaoning Normal University, Dalian, 116029 People’s Republic of China; College of Pharmacy, Dalian Medical University, Dalian, 116600 People’s Republic of China; Liaoning University of Traditional Chinese Medicine, Dalian, 110032 People’s Republic of China; Department of Orthopaedic Surgery, First Affiliated Hospital of Dalian Medical University, Dalian, 110032 People’s Republic of China

**Keywords:** *Fructus Chebulae Immaturus*, Total tannin, Ethyl gallate, Antibacterial, Electron microscopy

## Abstract

**Background:**

To extract, purify, and identify the effective constituents of aqueous extract of *Fructus Chebulae Immaturus*, and analyze the bactericidal effects of total tannins.

**Methods:**

Preparative thin layer chromatography and semi-preparative high performance liquid chromatography were used to isolate and purify the total tannin fraction. ^1^H- and ^13^C- NMR spectroscopy were used to elucidate compound structures. The antibacterial activities of total tannins and ethyl gallate on *Klebsiella pneumoniae* (KP) and *Staphylococcus aureus* (SA) were determined through minimum inhibitory concentration and minimum bactericidal concentration assays. Their antibacterial mechanisms of action were explored by transmission electron microscopy and scanning electron microscopy.

**Results:**

Five compounds were isolated: ellagic acid, ethyl gallate, arjugenin, β-sitosterol, and tri-n-butyl chebulate. Tri-n-butyl chebulate is a newly-reported compound. Total tannins and ethyl gallate both had favorable bactericidal effects against KP and SA.

**Conclusion:**

In vivo and in vitro pharmacodynamic experiment demonstrated that the effective components of *Fructus Chebulae Immaturus* possessed significant antibacterial effects, and were nontoxic and safe.

**Trial registration:**

No results of a health care intervention on human participants

## Background

*Fructus Chebulae Immaturus*, also known as Zangqingguo, is the immature fruit of *Terminalia chebula* Retz. in the family Combretaceae. The mature fruit *Fructus Chebulae*, known as Helile, was originally recorded in the “Synopsis of Golden Chamber.” Nonlignified young fruits of *Terminalia chebula* are harvested, boiled in water for 2–3 min, and dried to yield *Fructus Chebulae Immaturus. Fructus Chebulae Immaturus* can clear heat, promote fluid generation, detoxify, and treat diarrhea. The fruits are used to treat laryngitis, bacillary dysentery, and tonsillitis. The “Newly Revised Materia Medica” recorded it as bitter, warm, and nontoxic, and effective in the treatment of cold qi, heart, and abdominal distention and fullness, and dysphagia. According to the “National Herbal Compendium,” it can treat diarrhea, arrest bleeding, restrain lungs, resolve phlegm, and is used in the treatment of chronic enteritis, chronic bronchitis, asthma, chronic laryngitis, ulcers, hemafecia, and rectocele [1].

Burns and scalds damage normal cells, which leads to decreased immunity. In complex clinical environments, wound surfaces are highly prone to bacterial infections, which affects the healing of wound surfaces [2, 3]. When wounds are infected, especially by drug-resistant bacteria, we face enormous challenges in clinical practice [4, 5]. Pharmaceutical specialists have been trying to find an antibacterial drug that does not easily produce resistance and promotes wound surface healing, but the results are not very desirable [6, 7]. Some studies emphasize microsubstances, and focus on exact and single active monomers [8]. These studies have uncovered a large number of effective monomers from natural medicines, and resulted in several successful drugs. Therefore, we aimed to study *Fructus Chebulae Immaturus*, a traditional Chinese medicine, to find effective antibacterial compounds [9, 10].

In experiments investigating single active monomers, effective components are extracted, purified, and further enriched from plant material. This is followed by isolation and analysis of the chemical constituents, and the pharmacodynamic differences between the effective components and the monomer constituents are compared. This type of experiment greatly reduces workload and is cost-effective. Pharmacodynamic study of monomers reduces the influence of impurities, and makes experimental results more scientific and rigorous.

Compounds from *Fructus Chebulae Immaturus* and their antibacterial activities have not been widely reported. The antibacterial effects of total tannins, ethyl gallate, and tri-n-butyl chebulate on *Klebsiella pneumoniae* (KP) and *Staphylococcus aureus* (SA) were investigated and the antibacterial mechanism was preliminarily explored using transmission electron microscopy (TEM).

## Methods

### Instrument and reagents

U-3010 UV-VIS spectrophotometer (Hitachi, Japan); Bruker 400 MHZ and 500 MHZ NMR spectrometers (Bruker, Germany). Reagents were all analytical grade.

### Drugs

*Fructus Chebulae Immaturus* was purchased from Baidu Medicine Co., Ltd. (Dalian, China), and identified by Professor Wang Bing of the Department of Medicinal Plant, College of Pharmacy, Liaoning University of Traditional Chinese Medicine as the dried young fruits of *Terminalia chebula* Retz. A voucher specimen was deposited in a pharmacognosy laboratory with specimen number XT007. Total tannins, ethyl gallate, and tri-n-butyl chebulate were prepared preliminarily in the laboratory. Ethyl gallate had a purity of 96 %.

### Bacterial strains and media

KP (ATCC700603) and SA (ATCC25923) were provided by the College of Pharmacy of Dalian Medical University. Luria–Bertani (LB) liquid medium, LB agar (LBA) medium, nutrient broth (NB) medium, and NB agar (NBA) medium were all purchased from Hope Biotechnology Co., Ltd. (Qingdao, China).

### Identification of effective constituents of *Fructus Chebulae Immaturus* [11]

*Fructus Chebulae Immaturus* (10 g) was weighed, soaked in a 15-fold amount of water for 20 min, and extracted at 50 °C for 1 h. Then, the filtrate was filtered, and concentrated to 10 mL (1 g of crude drug per ml) by heating in a constant temperature water bath. Crude products of total tannins, total polysaccharides, and total saponins were all prepared in accordance with classical extraction methods, each of which were then concentrated to 10 ml (1 g of crude drug per ml).

A 5-cm-long, 1-cm-wide shallow trench was cut out in the middle of the solid medium, into which 1 ml *Fructus Chebulae Immaturus* aqueous extract, total tannin crude product, total polysaccharide crude product, and total saponin crude product were poured. The cultured bacteria were inoculated on both sides of the shallow trench, placed in a thermostatic shaker set at 37 °C, and statically cultured for 18 h. Results were then observed, and the length of the nonbacterial growth ring was recorded as “+” for 1–4 mm, “++” for 5–8 mm, “+++” for 9 mm or above, and “−” for no antibacterial effect.

### Inhibitory effects of drug-containing sera on KP and SA [12]

#### Animal grouping and collection of drug-containing sera

Mice were randomly divided into three groups: control group, *Fructus Chebulae Immaturus* aqueous extract group, and total tannin crude product group, n = 10 in each group. The mice were administered drugs intragastrically at a dose of 0.15–0.26 ml (1 g of crude drug per ml), which was converted according to the conversion formula of mouse and human medication doses. The control group was given 0.2 ml of normal saline twice daily for three consecutive days. All experimental procedures were approved by the Animal Research Ethics Committee of Dalian Medical University, Dalian, China (DMU10/02/23).

Before the last administration, mice were fasted from food and water, 0.5 h after administration. Blood was collected from the eyeball, centrifuged at 3000 rpm for 15 min, and the supernatant sera were aspirated. Sera were inactivated at 56 °C for 30 min, filtered through Millipore membrane to remove bacteria, and stored at −20 °C for later use.

#### *Bacteria classification and determination of bacteriostatic rate* [13]

Positive control group: cefoperazone sodium 5 μl + bacterial solution 95 μl; negative control group: serum of mice in the control group 5 μl + bacterial solution 95 μl; blank control group: culture medium 5 μl + bacterial solution 95 μl; sample group: serum of mice in the treatment group 5 μl + bacterial solution 95 μl. Solutions were cultured statically in 96-well plates in a constant temperature shaker set at 37 °C for 18–24 h. OD was measured at 620 nm with an enzyme-linked immunoabsorbent assay reader, and the inhibition rate of the sample was calculated.

#### Further isolation and purification of effective constituents

Total tannin crude product was obtained by chromatography on a silica gel column. Gradient elution was used with a chloroform-methanol system (with ascending polarity), and isolated by column chromatography with 300 ml as one fraction. Then, identical fractions were combined. The resulting crude extract was purified by preparative TLC and semi-preparative high-performance liquid chromatography to yield five compounds.

### Antibacterial effects of total tannins, ethyl gallate, and tri-n-butyl chebulate on KP

#### Determination of minimum inhibitory concentration (MIC) and minimum bactericidal concentration (MBC)

Drug solutions were prepared at concentrations of 10, 5, 2.5, 1.25, 0.625, 0.313, 0.156, 0.078, and 0.039 mg/ml, and 1 ml of each solution was added to test tubes. Another test tube was filled with 1 ml of culture medium. 1 ml of 10^6^ CFU/ml bacterial solution was added to test tubes. After culturing at 37 °C for 18 h, clarity of each tube was observed, and the lowest concentration of drug at which the solution was clarified was regarded the MIC of the test sample. Culture medium (100 μl) in the clarified test tube was aspirated and applied onto LBA medium, after culturing at 37 °C for 18 h. The lowest concentration of drug at which no colonies appeared on the plate was considered the MBC of the test sample.

#### Determination of bactericidal curve of ethyl gallate on KP

Bactericidal curves were measured by test tube method, with a final concentration of bacterial solution of 5 × 10^5^ CFU/ml. Test samples with the concentrations 4MIC, 2MIC, MIC, and 1/2MIC, and a fifth blank broth tube were used. Bacterial solutions were taken at 0, 2, 4, 6, 8, 12, 18, and 24 h. After spreading onto the plates, the number of viable cells of each concentration of test sample at each time was recorded, and the bactericidal graphs were plotted.

### TEM and SEM determination [14]

#### Selection of time points

Bacterial solution (30 ml) that was diluted 10^3^ times was placed in culture flasks, and cultured for three 12 h, 18 h, or 24 h. After separately centrifuging the bacterial solutions, they were processed into TEM samples for observation of bacterial growth at various times, and the time points of the experimental bacteria were determined.

#### TEM determination

Concentration of total tannins was prepared as 1/2MIC, and the concentration of ethyl gallate was prepared as 1/2MIC and MIC. After separately adding to the pre-diluted bacterial solution and cultured for 18 h, samples were processed into TEM samples, and observed under a 20Kx electron microscope.

#### SEM determination

Concentration of total tannins was prepared as MIC, and the concentration of ethyl gallate was prepared as MIC and 2MIC. After separately adding to the pre-diluted bacterial broth and cultured for 18 h, samples were made into SEM samples as per the SEM requirements, and observed under a 10Kx electron microscope.

## Results

### Antibacterial effects of various extractive fractions of *Fructus Chebulae Immaturus* on KP and SA

As shown in Table 1, *Fructus Chebulae Immaturus* aqueous extract and total tannin crude product had inhibitory effects on KP and SA. The inhibitory effect was especially prominent in the total tannin crude product against KP. However, total polysaccharide crude product and total saponin crude product had no inhibitory effect on either of the strains.

**Table 1:** Results of antibacterial effects of various extractive fractions of *Fructus Chebulae Immaturus* on KP and SA

	Antibacterial effect	
	KP	SA
*Fructus Chebulae Immaturus* aqueous extract	++	++
Total tannin crude product	+++	++
Total polysaccharide crude product	−	−
Total saponin crude product	−	−

“+” indicates 1–4 mm, “++” indicates 5–8 mm, “+++” indicates 9 mm or above, and “−” indicates no antibacterial effect

### Inhibitory effects of drug-containing sera on KP and SA

As shown in Table 2, sera of mice in the negative control group had no inhibitory effect on either of the two strains. Mouse sera containing *Fructus Chebulae Immaturus* aqueous extract and total tannin crude product had relatively strong inhibitory effects on both strains. The inhibitory effects were better against KP than SA, with the inhibition rate of total tannin crude product on KP reaching 66.26 %.

**Table 2:** Inhibition rates of *Fructus Chebulae Immaturus*-containing sera on KP and SA

	Inhibition rate %	
	KP	SA
Positive control group	80.32	73.13
Negative control group	-	-
Blank control group	-	-
*Fructus Chebulae* Immaturus aqueous extract	56.32	42.14
Total tannin crude product	66.26	58.55

### Structural identification of compounds

Compound 1: white granular crystals (methanol), m.p.: 129–133 °C. HR-MS [M + Na] + *m/z*: 547.2150. Ferric chloride-potassium ferricyanide reaction positive, three active hydrogen proton signals δ9.79, 9.55, 9.21 (each 1H) were observed in the 1H-NMR (DMSO-d6, 400 MHz), suggesting the presence of phenolic hydroxyl groups in the structure. δ6.92 (1H, s) was an isolated aromatic proton signal. δ5.27 (1H, d, J = 0.8 Hz) was a hydrogen signal on oxygenated carbon. Two groups of proton signals were observed on saturated carbon δ3.70 (1H, dd, J1 = 0.8 Hz, J2 = 7.6 Hz), 3.07 (1H, m). A group of geminally coupled methylene proton signals were observed at δ2.76 (1H, dd, J1 = 16.8, J2 = 10.8 Hz) and 2.35 (1H, dd, J1 = 16.8, J2 = 4.0 Hz). In addition, the high field region also showed three methylene proton signals on oxygenated carbon at δ3.99, 3.96 (each 2H, m), and 3.90 (2H, t, J = 2.4 Hz). Six groups of saturated methylene proton signals at δ1.51, 1.47, 1.35, 1.29, 1.25, and 1.10 (each 2H, m). Three groups of methyl proton signals were seen at δ0.84–0.87 (6H, m) and 0.75 (3H, t, J = 7.2 Hz). 13C-NMR (DMSO-d6, 100 MHz) gave a total of 26 carbon signals, of which δ172.4, 171.0, 169.3, and 162.9 were four carbonyl carbon signals; δ145.5, 142.7, 138.8, 115.7, 114.6, and 107.5 were a group of carbon signals on aromatic ring, and 76.4, 64.9, 64.4, and 63.8 were four oxygenated carbon signals. The high field region also showed twelve saturated carbon signals. Through the above analysis, and combined with the preliminary studies, the compound was generally identified as a chebulic acid derivative. The protons on each carbon signal were assigned by HSQC spectral analysis.

**Fig. 1:**
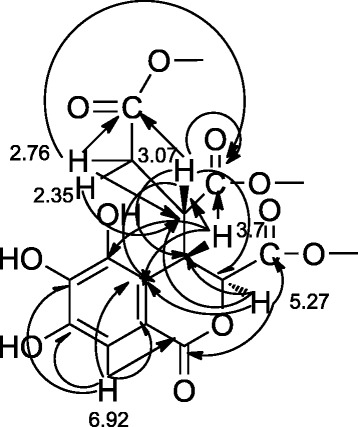
Diagram of parent nucleus fragment of chebulic acid

In the HMBC spectrum, the aromatic protons of δ6.92 (1H, s) showed long-range correlation with δ142.7, 138.8, 115.7, and 114.6, and the carbonyl carbon signals of δ162.9. Protons on the oxygenated carbon of 5.27 (1H, d, J = 0.8 Hz) presented long-range correlation with the aromatic carbon signals of δ115.7 and the carbonyl carbon signals of δ169.3 and 162.9, and its long-range correlation signals with δ43.2 and 35.7 were also observed. Proton signals of δ3.70 (1H, dd, J1 = 0.8 Hz, J2 = 7.6 Hz) had long-range correlation with the carbonyl carbon signals of δ172.4, aromatic carbon signals of δ145.5, 115.7, and 114.6, and saturated carbon signals of δ43.2 and 33.7. Proton signals of δ3.07 (1H, m) presented long-range correlation with carbonyl carbon signals of δ172.4, 171.0, aromatic carbon signals of δ115.7, oxygenated carbon signals of δ76.4, and saturated carbon signals of δ35.7. Geminally coupled methylene proton signals δ2.76 (1H, dd, J1 = 16.8, J2 = 10.8 Hz) and 2.35 (1H, dd, J1 = 16.8, J2 = 4.0 Hz) all had long-range association with carbonyl carbon signals of δ172.4, 171.0, and saturated carbon signals of δ43.2 and 35.7. Through the above long-range correlation information, the parent nucleus fragment of chebulic acid was identified as shown in Fig. 1.

**Fig. 2:**
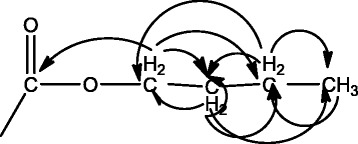
Diagram of n-butyl structure fragment

Further observation of HMBC spectrum found the long-range correlation of three groups of methylene proton signals on oxygenated carbon at δ3.99, 3.96, and 3.90 with three carbonyl carbon signals of δ172.4, 171.0, and 169.3, and six methylene signals of δ30.0, 29.9, 18.5, and 18.2. Three groups of saturated methylene proton signals at δ1.51, 1.47, and 1.35 (each 2H, m) presented long-range correlation with oxygenated carbon signals of δ64.9, 63.8, methylene carbon signals of δ18.5, 18.2, and methyl carbon signals of δ13.5, 13.3. The other three groups of saturated methylene proton signals δ1.29, 1.25, and 1.10 (each 2H, m) had long-range correlation with oxygenated carbon signals of δ64.9 and 63.8, methylene carbon signals of δ30.0 and 29.9, and methyl carbon signals of δ13.5 and 13.3. Three groups of methyl proton signals δ0.84–0.87 (6H, m) and 0.75 (3H, t, J = 7.2 Hz) showed long-range correlation with methylene carbon signals δ30.0, 29.9, 18.5, and 18.2. Through these series of long-range correlation signals, the presence of three n-butyl structure fragments were identified in the structure, which were connected to the three carboxyl groups of chebulic acid, as shown in Fig. 2.

**Fig. 3:**
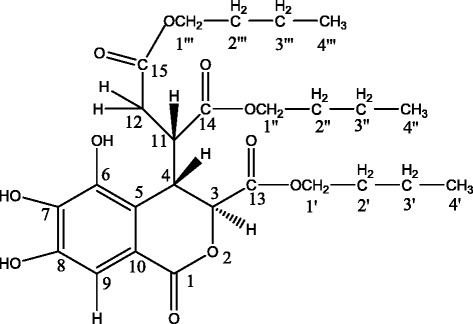
Structure of tri-n-butyl chebulate

Based on the above analysis, the two fragments were ligated together to give the complete structure of the compound, as shown in Fig. 3 and Table 3. Therefore, the compound was identified as tri-n-butyl chebulate. The compound was not previously reported, so it was confirmed as a new compound.

**Table 3:** ^1^H,^13^C-NMR spectral data of tri-n-butyl chebulate [in DMSO-d6, TMS]

No.	δ H	δ C	No.	δ H	δ C
1		162.9	1′	3.96 (2H, m)	64.9
2			2′	1.47 (2H, m)^a^	30.0^d^
3	5.27 (1H, d, J = 0.8 Hz)	76.4	3′	1.25 (2H, m)^b^	18.5^e^
4	3.70 (1H, dd, J = 0.8 Hz, 7.6 Hz)	35.7	4′	0.84 (3H, m)^a^	13.5^f^
5		115.7	1″	3.99 (2H, m)	64.9
6		145.5	2″	1.51 (2H, m)^a^	30.0^d^
7		138.8	3″	1.29 (2H, m)^b^	18.5^e^
8		142.7	4″	0.87 (3H, m)^c^	13.5^f^
9	6.92 (1H, s)	107.5	1‴	3.90 (2H, t, J = 2.4 Hz)	63.8
10		114.6	2‴	1.35 (2H, m)^c^	29.9^d^
11	3.07 (1H, m)	43.2	3‴	1.10 (2H, m)^b^	18.2^e^
12	2.76 (1H, dd, J = 16.8, 10.8 Hz)	33.7	4‴	0.75 (3H, t, J = 7.2 Hz)^c^	13.3^f^
	2.35 (1H, dd, J = 16.8, 4.0 Hz)				
13		169.3			
14		172.4			
15		171.0			

^a,^ ^b,^ ^c,^ ^d,^ ^e,^ ^f:^ overlap, can be exchanged

**Table 4:** MIC and MBC of total tannins, ethyl gallate, and tri-n-butyl chebulate against KP

NO	MIC (mg/ml)	MBC (mg/ml)
Total tannins	0.3125	0.625
Ethyl gallate	0.156	0.3125
Tri-n-butyl chebulate	1.25	5

**Fig. 4:**
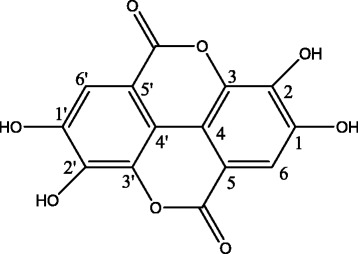
Structure of ellagic acid

Compound 2: brown crystals (methanol). Mp: 349–351 °C; ^1^H-NMR (400 MHz, DMSO-d6) δ: 8.16 (2H, s, H-6, 6′); ^13^C-NMR (C_5_D_5_N, 100 MHz), δ: 160.6 (C = O), 149.3 (C-3, 3′), 141.9 (C-1, 1′), 137.8 (C-2, 2′), 113.5 (C-4, 4′), 111.8 (C-5, 5′), 108.7 (C-6, 6′). The above analysis was consistent with the literature [9], so compound 2 was identified as ellagic acid, and its structure is shown in Fig. 4.

**Fig. 5:**
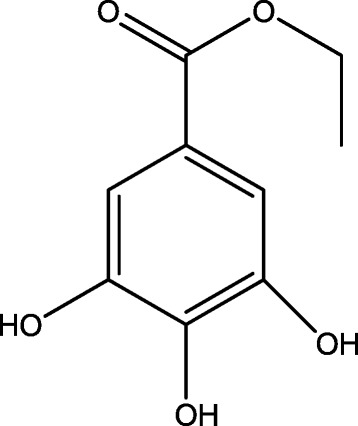
Structure of ethyl gallate

Compound 3: white needle crystals (chloroform). Mp: 155–157 °C. ^1^H-NMR (400 MHz, DMSO-d6) δ: 1.27 (3H, t, J = 5.6 Hz), 9.23 (2H, s, 3-OH, 5-OH), 8.90 (1H, s, 4-OH), 6.95 (2H, s, H-2, 6), 4.20 (2H, q, J =5.6 Hz, CH_2_), 1.27 (3H, t, J =5.6 Hz, CH_3_); ^13^C-NMR (DMSO-d6, 100 MHz) δ: 14.2 (CH_3_), 59.9 (CH_2_), 108.4 (C-2, 6), 119.6 (C-1), 138.3 (C-4), 145.5 (C-3, 5), 165.8 (-CO-). The above analysis was consistent with the literature [15], so compound 3 was identified as ethyl gallate, and its structure is shown in Fig. 5.

**Fig. 6:**
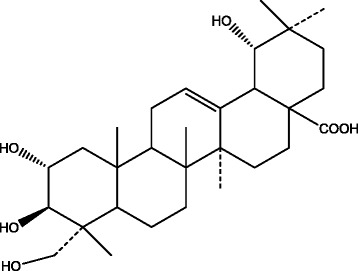
Structure of arjugenin

Compound 4: white powdery crystals (methanol). Mp: 292–294 °C. ^1^H-NMR (C_5_D_5_N, 400 MHz) δ: 1.09, 1.11, 1.13, 1.20, 1.59, 2.13 (each 3H, s, 6 × CH_3_), 3.63 (2H, s, CH_2_OH), 3.74 (1H, d, J = 10.8 Hz, H-19), 4.23 (1H, m, H-2), 4.28 (1H, m, H-3), 5.57 (1H, br.s, H-12); ^13^C-NMR (C_5_D_5_N, 100 MHz) δ: 48.0 (C-1), 69.4 (C-2), 78.8 (C-3), 44.2 (C-4), 48.6 (C-5), 19.2 (C-6), 34.1 (C-7), 40.6 (C-8), 49.0 (C-9), 39.1 (C-10), 29.3 (C-11), 123.7 (C-12), 145.4 (C-13), 42.7 (C-14), 29.6 (C-15), 24.8 (C-16), 46.5 (C-17), 45.3 (C-18), 81.7 (C-19), 36.2 (C-20), 28.8 (C-21), 33.5 (C-22), 67.0 (C-23), 14.8 (C-24), 18.1 (C-25), 17.8 (C-26), 25.3 (C-27), 181.4 (C-28), 29.6 (C-29), 25.3 (C-30). The above data were consistent with the literature [16], so compound 4 was identified as 2α,3β,19α,23-tetrahydroxy-olean-12-en-28-oic acid, or arjugenin, and its structure is shown in Fig. 6.

**Fig. 7:**
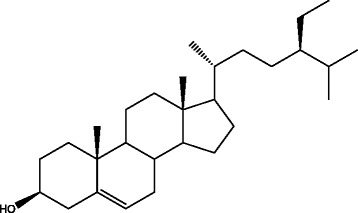
Structure of β-sitosterol

Compound 5: Colorless needle crystals (petroleum ether-acetone). Mp: 136–137 °C. ^1^H-NMR (C_5_D_5_N, 400 MHz)δ: 0.71 (3H, s, H-18), 1.09 (3H, s, H-19), 1.02 (3H, d, J = 6.4 Hz, H-21), 0.91 (3H, br.d, J = 1.2 Hz, H-26), 0.89 (3H, br.d, J = 1.2 Hz, H-27), 0.88 (3H, br.d, H-29), 5.44 (1H, br.d, J = 4.4 Hz, H-6), 3.87 (1H, m, H-3); ^13^C-NMR (100 MHz, C_5_D_5_N)δ: 38.3 (C-1), 32.7 (C-2), 71.8 (C-3), 46.6 (C-4), 142.5 (C-5), 121.7 (C-6), 34.7 (C-7), 32.7 (C-8), 51.0 (C-9), 37.4 (C-10), 21.9 (C-11), 40.5 (C-12), 43.0 (C-13), 57.4 (C-14), 25.0 (C-15), 29.0 (C-16), 56.8 (C-17), 12.7 (C-18), 20.1 (C-19), 36.9 (C-20), 19.7 (C-21), 33.1 (C-22), 23.9 (C-23), 30.0 (C-24), 29.1 (C-25), 20.5 (C-26), 19.5 (C-27), 26.9 (C-28), 12.5 (C-29). The above data were consistent with the spectral data of β-sitosterol reported in the literature [17], so compound 5 was identified as β-sitosterol as shown in Fig. 7.

**Fig. 8:**
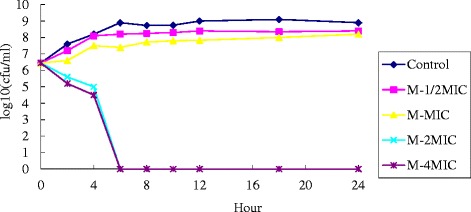
Bactericidal curve of ethyl gallate against KP

### Results of MIC and MBC of total tannins, ethyl gallate, and tri-n-butyl chebulate against KP

MIC and MBC of ethyl gallate were both lower than those of total tannins, which indicates that ethyl gallate has antibacterial activity. MIC and MBC of tri-n-butyl chebulate were both higher than those of total tannins and ethyl gallate, indicating that its inhibitory effect on KP was weaker, but the experiments demonstrated that tri-n-butyl chebulate still had some antibacterial effects (Results showed in Table 4).

### Results of bactericidal curve of ethyl gallate against KP

As can be seen from the bactericidal curve in Fig 8, when the drug concentration was 2MIC, it could kill almost all bacteria at around 6 h. Moreover, the bactericidal action did not increase with increasing drug concentration, indicating that the bactericidal effect of ethyl gallate against KP was time-dependent.

### TEM results

**Fig. 9:**
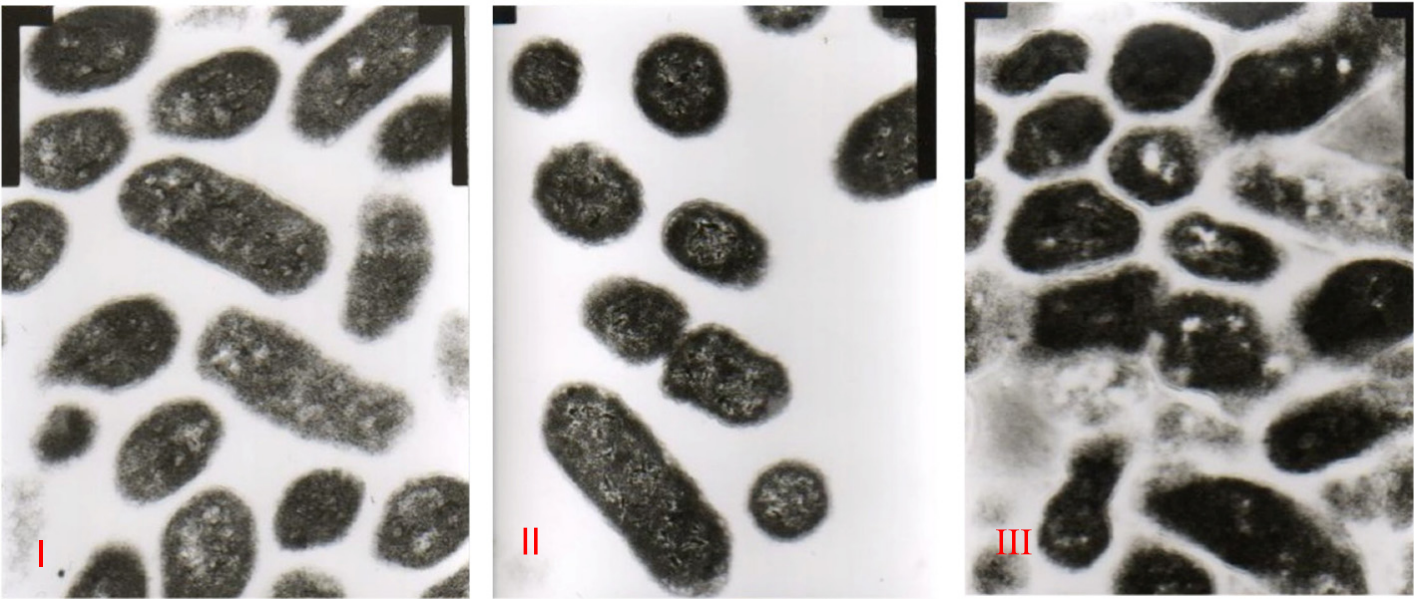
TEM of normal bacterial morphology. I: 12 h; II: 18 h; III: 24 h

Under high magnification (20Kx) microscope, after 12 h, the bacterial cell walls and membranes were relatively intact, tightly bound, and the cytoplasm was relatively homogeneous (Fig. 9 panel I). After 18 h, the bacterial cell walls and membranes were intact, smooth, tightly bound, the cytoplasm was homogeneous, abundant, and filled with ribosomes, and midbodies were present (Fig. 9 panel II). At 24 h, part of the cell walls and membranes were ruptured, and there were more dead bacteria (Fig. 9 panel III). These results indicate that normal bacteria were relatively healthy at 12 h, and healthiest at 18 h. To ensure the best results for the antibacterial experiments, the bacteria cultured for 18 h were selected as the test bacteria.

**Fig. 10:**
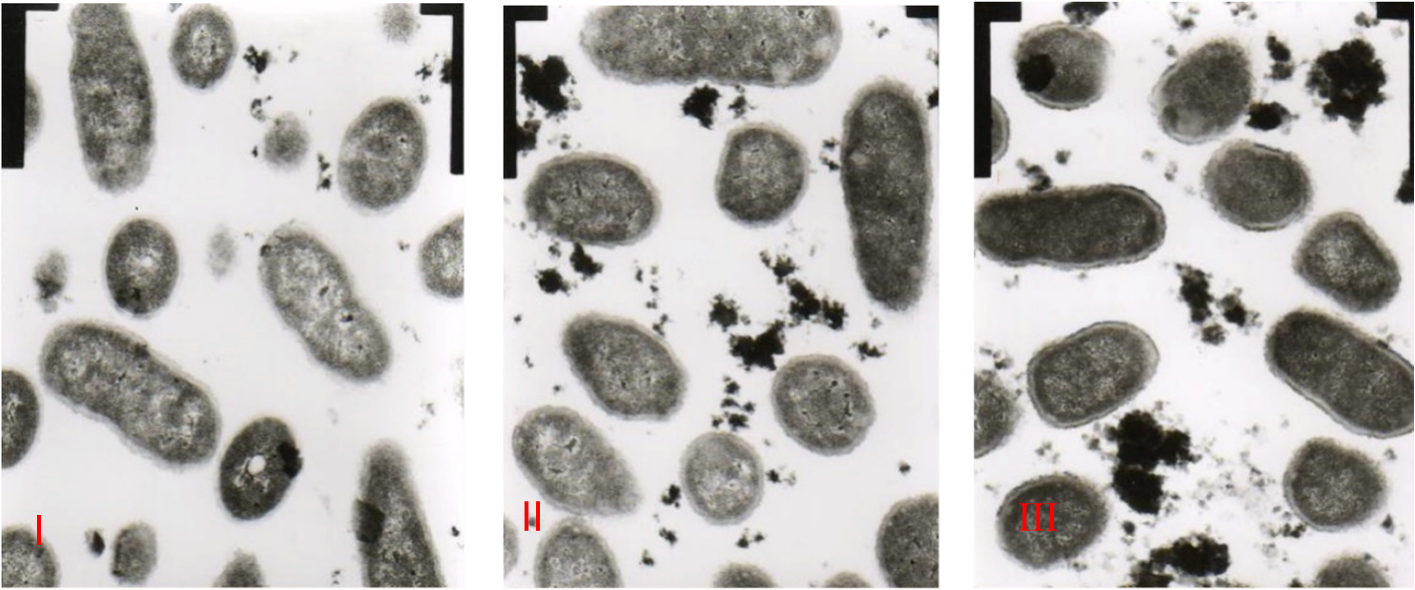
TEM. I: 1/2MIC total tannins; II: 1/2MIC ethyl gallate; III: MIC ethyl gallate

The results showed that, 18 h after addition of 1/2MIC total tannins, bacteria were in long strips or amorphous, with evident plasmolysis. Bacterial cell walls were severely damaged, and the wall boundaries were not clearly distinguished, and were intermittent. The cytoplasms were loose, and cell structures were in a loose form (Fig. 10 panel I). After the addition of 1/2MIC ethyl gallate, the bacterial cytoplasm gathered into a mass, with a cavity in the middle. A large number of bacterial cell walls were ruptured along with membranes, and massive leakage of cell contents were observed at the rupture sites (Fig. 10 panel II). The state of bacteria with MIC ethyl gallate was similar to that of 1/2 MIC ethyl gallate (Fig. 10 panel III).

### SEM results

**Fig. 11:**
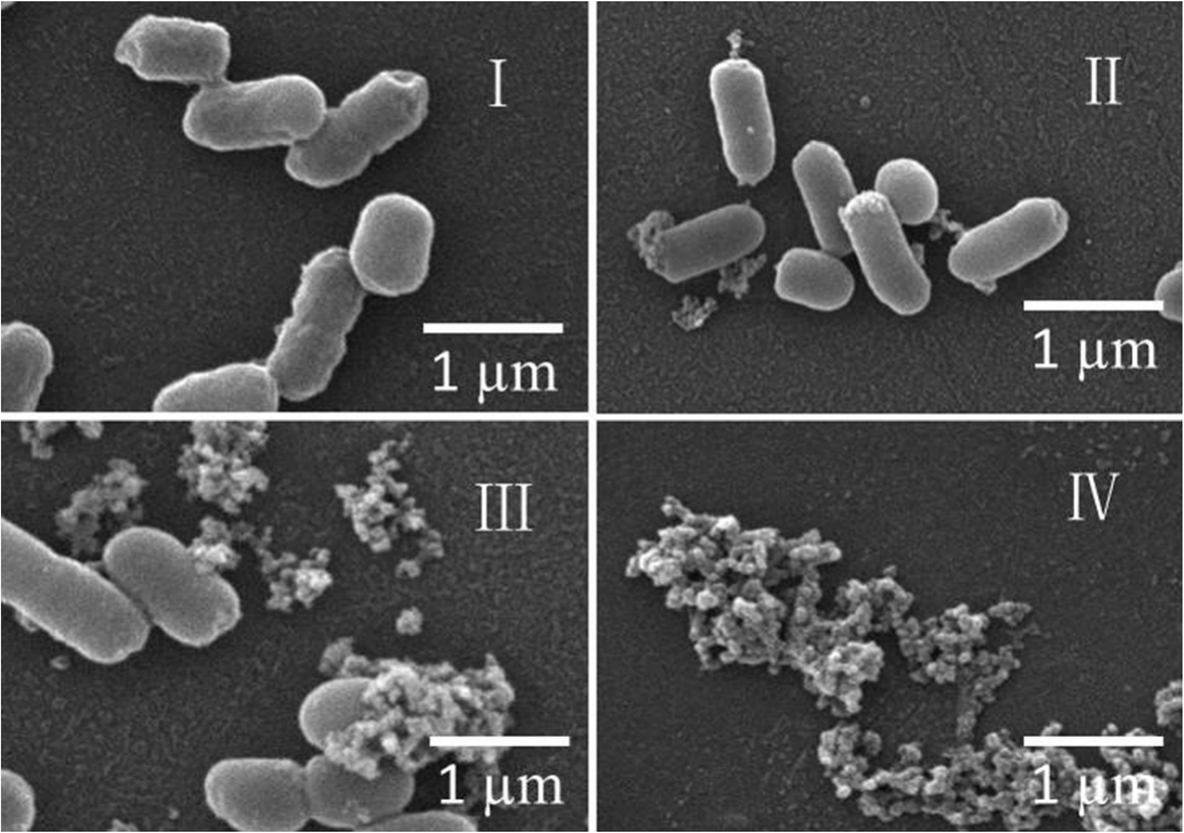
SEM. I: normal bacterial morphology; II: MIC total tannins; III: MIC ethyl gallate; IV: 2MIC ethyl gallate

SEM observation found that normal KP had a smooth surface, plump appearance, and good refraction (Fig. 11 panel I). After addition of MIC total tannins, the bacterial morphology changed markedly compared with the control group. The majority of bacteria were adhered to each other, and some were shrunken and deformed (Fig. 11 panel II). The morphology of bacteria given MIC ethyl gallate was similar to that of MIC total tannins. The majority of bacteria were adhered to each other, some were shrunken, dried, bent, and deformed, and some had significant pitting, cavities, or amorphous protruding structures. A few bacteria were lysed (Fig. 11 panel III). When the concentration of ethyl gallate was increased to 2MIC, significant lysis was observed in all bacteria. Cell walls and membranes were ruptured completely, and the bacteria were dead (Fig. 11 panel IV). Therefore, the effective components and ethyl gallate both had significant inhibitory effects on KP at MIC concentration.

## Discussion

In this experiment, the antibacterial activities of various components of *Fructus Chebulae Immaturus* were screened, and effective antibacterial activity of total tannins was observed. The effective fractions also had good antibacterial effects in animals. The antibacterial effects were stronger against KP than SA.

To further identify the monomer compounds in the effective fractions, a bacteriostatic experiment was conducted on each fraction of total tannin group from the column chromatography, and ethyl gallate and tri-n-butyl chebulate were found to have better inhibitory effects. Three additional compounds, ellagic acid, arjugenin, and β-sitosterol, were also isolated from other fractions.

Further study showed that tri-n-butyl chebulate had a weaker antibacterial effect on KP than ethyl gallate. Nevertheless, it still possessed some antibacterial effects and was contained in the effective fraction. Future study will explore the antibacterial effect of tri-n-butyl chebulate combined with ethyl gallate, and further identify the molecular mechanisms of action.

During the experiment, the bactericidal curve of ethyl gallate on KP was plotted, and time-dependency of its bactericidal effect was demonstrated. The bactericidal mechanism of ethyl gallate was preliminarily investigated by TEM and SEM, and found to be via rupture of bacterial cell membranes and cell swelling.

The innovative points of this experiment were the identification of components with antibacterial activity in *Fructus Chebulae Immaturus*, isolation of a compound with better antibacterial activity from total tannins (ethyl gallate), and isolation and identification of a new compound from *Fructus Chebulae Immaturus*, tri-n-butyl chebulate. The experiment demonstrated that ethyl gallate has a favorable anti-KP effect, and we hypothesize that the antibacterial role was played mainly by these compounds in *Fructus Chebulae Immaturus*. Determining the content of each compound in plants, and how to conveniently isolate them were experimental limitations.

Experimental results showed that the antibacterial effect of ethyl gallate was similar or superior to total tannin crude product. Therefore, ethyl gallate was one of effective constituents in total tannin crude product of *Fructus Chebulae Immaturus* with antibacterial activity. Because of its relatively low content in the total tannin crude product, conveniently and efficiently extracting ethyl gallate remains a problem.

This paper preliminarily investigated the antibacterial mechanism of ethyl gallate by TEM and SEM, without any in-depth analysis. Further studies focusing on the molecular mechanisms are required.

## Conclusions

In vivo and in vitro pharmacodynamic experiment demonstrated that the effective components of Fructus Chebulae Immaturus possessed significant antibacterial effects, and are nontoxic and safe. The study of their antibacterial mechanisms using electron microscopy techniques allowed analysis of the antibacterial mechanisms from a morphological point of view.

## Abbreviations

KP, Klebsiella pneumoniae; MBC, minimum bactericidal concentration; MIC, minimum inhibitory concentration; SA, Staphylococcus aureus; SEM, scanning electron microscope; TEM, transmission electron microscopy; TLC, thin layer chromatography
